# Endoscopy works during the pandemic of coronavirus COVID-19: recommendations by the Chinese Society of Digestive Endoscopy

**DOI:** 10.1177/2050640620930632

**Published:** 2020-07-02

**Authors:** Ningli Chai, Zhechuan Mei, Wengang Zhang, Chen Du, Xiangyao Wang, Longsong Li, Yan Ma, Jiale Zou, Xiaowei Tang, Nanjun Wang, Jiancong Feng, Enqiang Linghu

**Affiliations:** 1 Department of Gastroenterology, Chinese PLA General Hospital, Beijing, China; 2 Department of Gastroenterology, The Second Affiliated Hospital of Chongqing Medical University, Chongqing, China

**Keywords:** Endoscopy, colonoscopy, epidemiology, surgery, endoscopic ultrasound

## Abstract

Since December 2019, a novel coronavirus disease, COVID-19, has occurred in China and has spread around the world rapidly. As an acute respiratory infectious disease, COVID-19 has been included in type B infectious diseases and managed according to the standard of type A infectious disease in China. Given the high risk of COVID-19 infection during endoscopic procedures via an airborne route, the Chinese Society of Digestive Endoscopy issued a series of recommendations to guide the endoscopy works in China during the pandemic. To the best of our knowledge, no new infectious case of COVID-19 resulting from endoscopic procedures has been reported in China to date. Here, these recommendations are integrated to provide guidance about the prevention of COVID-19 for endoscopists. The recommendations include advice about postponing non-urgent endoscopies, excluding the possibility of COVID-19 in patients undergoing endoscopy, protection of medical staff from coronavirus infection, and cleaning of endoscopy centres.

## Background

COVID-19 was defined as a cluster of respiratory illnesses induced by the infection of the 2019 novel coronavirus (2019-nCoV).^1^ Since the outbreak of COVID-19 in Wuhan in December 2019, it has spread around the world rapidly. As an acute respiratory infectious disease, the disease has been included in type B infectious diseases and managed according to the standard of type A infectious disease, the highest level of infectious disease in China. The main transmission route of 2019-nCoV is respiratory droplets and close contact, and it is possible to be infected with 2019-nCoV for people exposed to an occlusive environment with high concentration aerosols for a long time; moreover, feces might also lead to 2019-nCoV infection via aerosols or close contact.^2^ Therefore, endoscopy was considered as a risky medical operation which might lead to 2019-nCoV infection. Since the initial stage of the outbreak of COVID-19 in China, the Chinese Society of Digestive Endoscopy (CSDE) has issued a series of recommendation documents to guide the endoscopy works, and achieved satisfactory results. To the best of our knowledge, no new infectious case of COVID-19 resulting from endoscopic procedures has been reported in China to date. Hence, we provide our experience to endoscopists all over the world under such challenging situations of the prevention and control of COVID-19.

## General principles

Selective endoscopic procedures are suggested for outpatients that do not have to receive endoscopic procedures immediately.

For patients that need to undergo endoscopic procedures as soon as possible, COVID-19 infection should be excluded initially by chest computed tomography (CT) results, polymerase chain reaction (PCR) results and personal history.

For patients infected with COVID-19, pneumonia should be treated at special medical institutions first; and if patients have to receive endoscopic procedures immediately on account of medical conditions, endoscopic procedures should be conducted in particular wards that have the ability to prevent COVID-19 infection.

## Preventive measures

First, doctors can provide education for patients via online and offline approaches, and tell them the potential risks of COVID-19 infection during endoscopic procedures. Selective endoscopic procedures are suggested for outpatients without urgent/emergent situations and malignant tumours, and we have provided educational material for patients from the CSDE in Supplement 1, namely *The CSDE Proposal for the Patients in the Pandemic Area of COVID-19* (see Supplementary material).

Second, endoscopic centres can undertake training activities for outpatient and consultant doctors, and help them master the protective knowledge. 2019-nCoV infection should be excluded initially by chest CT results, PCR results and personal history for patients that need to receive endoscopic procedures as soon as possible, such as a foreign body in the digestive tract, oesophageal and gastric varices bleeding, severe peptic ulcer bleeding, biliary tract obstruction, malignant tumours and so on. Of note, endoscopists can make a decision according to specific circumstances, if patients with urgent/emergent situations are not able to provide timely CT and PCR results; and endoscopic centres should pay close attention to all medical staff performing endoscopic procedures for patients without exact CT and PCR results. We recommend that the chest CT examinations and PCR tests should be performed in the last 3 days, and intraday results are optimal. We provide the material for recording information of patients from the CSDE in Supplement 2, namely *The CSDE Recommendation of Preliminary Screening Questionnaire for the Admission of Patients in COVID-19 Pandemic Area* (see Supplementary material). If endoscopic procedures have to be performed for patients infected with 2019-nCoV immediately on account of medical conditions, endoscopic doctors should fully communicate with respiratory or intensive care unit doctors; and endoscopic procedures should be conducted in particular wards that have the ability to prevent 2019-nCoV infection. Besides, for patients recovering from 2019-nCoV infection, endoscopic procedures could be conducted for them if necessary in a short time, and related proof material should be provided.

Third, endoscopic centres can set aside some special rooms for endoscopic procedures during the COVID-19 pandemic, and should conduct cleaning and disinfection work immediately after every procedure.

Fourth, endoscopic centres can make emergency plans. If patients with suspected infection with COVID-19 are found by outpatient and consultant doctors, endoscopic centres should submit the case report to the related medical institutions immediately, and the doctors who received the patients with suspected infection should be isolated and observed closely in proper ways. On the other hand, endoscopic centres can make intermittent follow-ups within 2 weeks after endoscopic operations for all patients receiving endoscopic procedures during the COVID-19 pandemic; and if patients with confirmed or suspected infection with COVID-19 are found, all related personnel that have had contact with these patients should be isolated and observed closely in proper ways.

Fifth, endoscopic centres can strengthen management for patients and family members, and take detailed measures as follows: set a one-way route in endoscopic centres, contain the number of waiting patients, enlarge spacing distance to at least 100 cm among waiting patients, and insist that patients and family members wash their hands before entering the waiting room and wear medical masks in the waiting process.

Of note, magnetic guide capsule examinations could separate the patients and operators in different rooms, and thus this technique might be an excellent alternative to endoscopic procedures for diagnosis gastroscopy and non-hematemesis haemorrhage.

## The workflow of endoscopic centres during the COVID-19 pandemic

**Figure 1.**
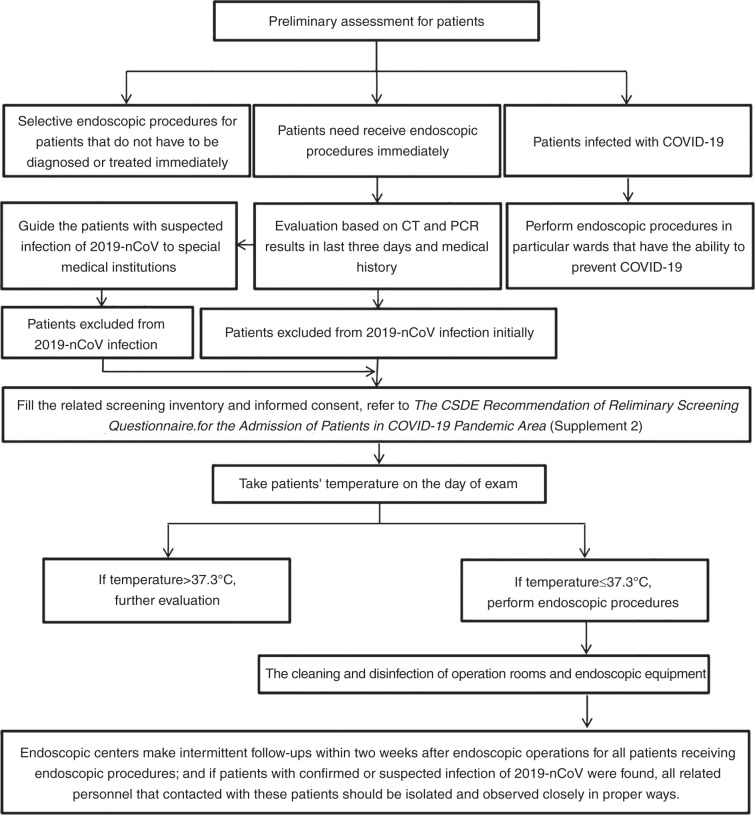
The workflow of an endoscopic centre during the COVID-19 pandemic.

The detailed workflow is shown in Figure 1.

### Personal protection for medical staff

First, medical staff of an endoscopic centre should have their temperature taken before going to work and leaving for work, and they should pay attention to the presence of some related symptoms including dry cough, fatigue and so on. If suspected symptoms of 2019-nCoV infection are found, medical staff should stop working immediately and be tested or evaluated further; and isolation measures ought to be taken if necessary.

Second, medical staff should wash their hands carefully according to related medical rules for at least 2 minutes every time, and alcohol-based hand rub is also recommended for more frequent hand disinfection.

Third, medical staff must wear medical masks in an endoscopic centre, and if the masks become contaminated, wet or are used for more than 4 hours, they should be replaced. Moreover, the number of medical staff including doctors, nurses and technicians in the operating room should be reduced to the minimum.

Fourth, medical staff should wear corresponding protective equipment according to different danger levels. For medical staff at the booking table, they should wear medical work clothes, isolation gowns (if conditions allow), N95 masks and medical work caps. For operating doctors, anaesthetists, nurses and technicians, they must wear medical work clothes, single-use medical protective clothing, anti-osmosis isolation gowns, medical protective caps, N95 masks, goggles or face shields, double medical gloves and shoe covers. Moreover, respirator masks should be provided for medical staff that perform tracheal intubation and sputum suction. For cleaning and disinfection workers, they should wear medical work clothes, single-use medical protective clothing, medical protective caps, N95 or higher level medical masks, goggles or face shields, medical gloves and long-sleeved rubber gloves; moreover, water-resistance isolation gowns and shoe covers should be provided if necessary. For medical staff delivering samples, they should wear medical work clothes, medical protective caps, rubber gloves, N95 or surgical masks; and a medical specimen box should be used during the delivery (Table 1).

**Table 1.** Personal protection for medical staff.

Personnel category	Hand disinfection 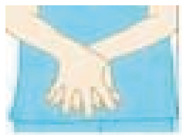	Medical work clothes 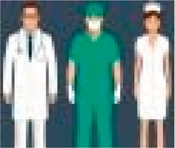	Surgical masks 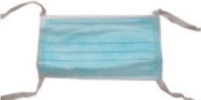	N95 or higher level medical masks 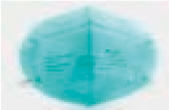	Goggles/face shields 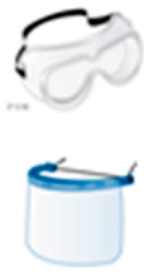	Rubber gloves 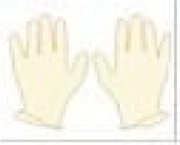	Isolation gowns 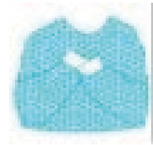	Medical protective clothing 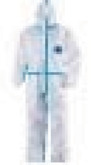	Medical protective caps 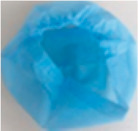	Shoe covers 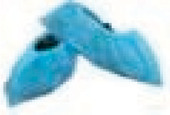
Medical staffat booking table	✓	✓		✓			✓ (If conditions allow)		✓	
Operating doctors, anaesthetists, nurses and technicians (in operating room)	✓	✓		✓	✓	✓	✓ (Anti-osmosis isolation gowns)	✓	✓	✓
Cleaning and disinfection workers	✓	✓		✓	✓	✓	✓ (Anti-osmosis isolation gowns)	✓	✓	✓
Medical staff delivering samples	✓	✓	✓	✓ (If conditions allow)		✓			✓	

Of note, respirator masks should be provided for medical staff that perform tracheal intubation and sputum suction.

Fifth, medical staff should take off the protective equipment according to the standard procedures.

Of note, endoscopic centres should provide surgical or N95 masks for patients receiving lower endoscopy procedures in order to protect both patients and medical staff from infection with 2019-nCoV. Moreover, given the high risk of 2019-nCoV infection during endoscopic procedures via an airborne route, including aspiration of oral and fecal material via endoscopes, we recommend the same medical protection level for upper and lower endoscopy.

## The cleaning and disinfection of endoscopic centres

### Disinfection of operating rooms

Each hospital should choose a suitable place for endoscopic procedures in compliance with the laws, guidelines and regulations for the prevention and control of COVID-19.^2–6^ We think that a negative-pressure room is the optimal solution, if conditions permit; and this point is in agreement with the suggestion of the American Society of Gastrointestinal Endoscopy (ASGE).^7^ Disinfection of the operating rooms is required immediately after every endoscopic procedure. It is recommended to use medical dynamic air disinfection equipment for continuous air disinfection in the endoscopy unit during the procedure. Terminal disinfection should be performed in accordance with the relevant standards.^6,8,9^ (a) Disinfection wipes, 75% alcohol and chlorine disinfectant can be used in the disinfection for the surface of the endoscopic equipment. The chlorine disinfectant should be wiped off with water after 30 minutes. (b) Chlorine disinfectant and chlorine dioxide disinfectant should be used for the disinfection of the ground and wiped off 30 minutes later. (c) Medical dynamic air disinfection equipment can be used for the disinfection of air in the endoscopy unit. The disinfection time should follow the manufacturer’s instructions. Other disinfection methods include the manual spray of 3% hydrogen peroxide, 5000 mg/L peroxyacetic acid, 500 mg/L chlorine dioxide, the use of atomisation or vaporisation hydrogen oxide steriliser and ultraviolet disinfection. (d) Open the window for sufficient ventilation after disinfection.

### Cleaning and disinfection of the endoscope

The endoscope should be cleaned and disinfected strictly according to the *Regulation for cleaning and disinfection technique of flexible endoscopes.*^10^ It is not recommended to perform bedside pretreatment for the endoscope.

The endoscope and reusable accessories should be put into a double-layered yellow medical waste bag and the related staff should seal the bag immediately after the operation. The 2019-nCoV logo must be put on the bag and then the related staff should transfer it to the endoscopy washroom.

Immerse all the endoscopes and reusable accessories immediately in 0.2–0.35% peroxyacetic acid or effective chlorine concentration 60 ± 10 mg/L acidified water for 5 minutes. It is recommended to use a syringe to fill the channels with detergent to ensure all the channels of the endoscope are fully soaked. Then clean and sterilise the endoscope according to specifications.

It is recommended to choose peracetic acid and chlorine-containing preparations as sterilant, other sterilants that meet the requirements can also be selected.

The related staff should clean and disinfect the cleaning tank, rinsing tank, perfusion device and cleaning brush thoroughly with chlorine-containing disinfectant, peroxyacetic acid or other disinfectants after the daily work in accordance with relevant national regulations, and the disinfectant should be wiped off after 30 minutes.

According to the related medical waste management regulations,^11^ hospitals must disinfect the sewage discharged from washing tanks, rinsing tanks or fully automatic washer-disinfectors with medical wastewater disinfection equipment before discharging them into the hospital sewage treatment system.

Medical waste should be packaged with double yellow medical waste bags marked with the logo of 2019-nCoV and disposed in accordance with the relevant regulations.^12,13^

## Conclusions

Currently, many countries are facing a very severe COVID-19 pandemic, and there exists a very high risk of 2019-nCoV infection during endoscopic procedures. Therefore, endoscopists must take appropriate measures immediately to prevent the potential spread of COVID-19 in endoscopic centres. The preventive works of COVID-19 in endoscopic centres has achieved preliminarily satisfactory results in China, and we hope this paper could provide a reference for endoscopists in the pandemic area of COVID-19 around the world.

## Supplemental Material

sj-pdf-1-ueg-10.1177_2050640620930632 - Supplemental material for Endoscopy works during the pandemic of coronavirus COVID-19: recommendations by the Chinese Society of Digestive Endoscopy Click here for additional data file. Supplemental material, sj-pdf-1-ueg-10.1177_2050640620930632 for Endoscopy works during the pandemic of coronavirus COVID-19: recommendations by the Chinese Society of Digestive Endoscopy by Ningli Chai, Zhechuan Mei, Wengang Zhang, Chen Du, Xiangyao Wang, Longsong Li, Yan Ma, Jiale Zou, Xiaowei Tang, Nanjun Wang, Jiancong Feng and Enqiang Linghu in United European Gastroenterology Journal
