# Supplementary material for: Endoscopy works during the pandemic of coronavirus COVID-19: recommendations by the Chinese Society of Digestive Endoscopy
Source: United European Gastroenterol J. 2020 Jul 2;8(7):798–803. doi: 10.1177/2050640620930632 (PMC7335943; doi:10.1177/2050640620930632)
Supplement: sj-pdf-1-ueg-10.1177_2050640620930632 - Supplemental material for Endoscopy works during the pandemic of coronavirus COVID-19: recommendations by the Chinese Society of Digestive Endoscopy [file sj-pdf-1-ueg-10.1177_2050640620930632.pdf]

Supplement 1

The CSDE Proposal for the Patients in the Pandemic area of COVID-19

Dear patients and their families:

The recent pandemic situation of COVID-19 is severe. The number of confirmed cases and deaths nationwide continues to rise, and there are a large number of potential virus carriers.

As public assembly occupancies, hospitals themselves are at risk of cross-infection. The transmission of 2019 novel coronavirus (2019-nCoV) is characterized by a long incubation period, strong infectivity and highly occultly. It can spread through multiple ways such as droplet transmission, and there is a possibility of fecal-oral transmission. The endoscopy procedures include appointment, waiting, examination, recovery and so on The large number of contacts and the inability of the patient to wear a mask during the endoscopic procedures enable the latent period patients to release virus. Moreover, asymptomatic patients in incubation period cannot be completely determined at this time. Therefore, you are highly likely to be infected with 2019-nCoV, if there is a latent patient in the same time as you receive endoscopy.

Therefore, in order to protect you and your families and reduce cross-infection, we recommend from a medical professional perspective: Unless there is an emergency situation, it is recommended that you do not receive endoscopic procedures during the outbreak, and go for examinations and treatments after the epidemic.

If your condition requires you to do endoscopic diagnosis and treatment immediately, please read the *Diagnosis and treatment of new coronavirus pneumonia from the national health commission of china* carefully with your family.

1. If you meet any of the following conditions, please take the initiative and inform the medical staffs truthfully, and follow their advice. After registering the information authentically and completely, visit the designated hospital according to the arrangement.

(1) Temperature above 37.3 degrees;

(2) Have travel or residence history in Wuhan or Hubei or other areas where local cases continue to spread within 2 weeks;

(3) Contacted with patients with fever or respiratory symptoms from Wuhan or Hubei, or other areas where local cases continue to spread within 2 weeks;

(4) There is a cluster disease in people around you, or these patients have epidemiological correlation with COVID-19;

(5) suffering from other suspicious symptoms such as cough, rhinorrhea, pharyngalgia, dyspnea, wheezing, chest tightness, stethalgia, night sweating, fatigue, diarrhea, etc.

2. Please keep a certain distance and wear a mask when you and your families come to see the doctor and talk with others. Please do not discard the used mask at

will, and put it into the designated special medical waste bin.

3. Visit the hospital with your real name and correct phone number, also bring ID card and health card with you.

4. For possible follow-up isolation, please read *The CSDE Recommendation of Reliminary Screening Questionnaire for the Admission of Patients in COVID-19 Pandemic Area* throughly and fill in the form truthfully when making an appointment or having an examination.

5. According to the current prevention and control requirements of COVID-19 of national and healthy institutions, you will be notified as soon as possible and required to be quarantined for two weeks, if a confirmed case is found in the patients who received endoscopy at the same time as you. The specific situation shall be implemented in accordance with relevant regulations.

Thank you for your understanding and cooperation!

We hereby declare that this proposal is recommended by the Chinese Society of Digestive Endoscopy (CSDE) for patients from the pandemic area of COVID-19. Please refer to the relevant medical institutions for specific implementation.

Chinese Society of Digestive Endoscopy (CSDE)
1 February 2020

Supplement 2

The CSDE Recommendation of Reliminary Screening Questionnaire for the Admission of Patients in COVID-19 Pandemic Area

（Please take necessary protective measures before communicating with medical staffs）

1. Are you coming from Wuhan or Hubei Province?

☐No ☐Yes (In accordance with hospital-related infection control regulations)

2. Have you been to Wuhan or Hubei Province recently?

☐No ☐Yes (Exact date ____________________

In accordance with hospital-related infection control regulations)

3. Have you recently contacted relevant personnel from Wuhan or Hubei Province?

☐No ☐Yes (Exact date ____________________

In accordance with hospital-related infection control regulations)

4. Is there a confirmed patient among the people you have recently contacted? Is there a cluster of cases?

☐No ☐Yes

5. If the answer for question 4 is "Yes", Please answer if you have been in contact with such patients?

☐No ☐Yes (Exact date ____________________

In accordance with hospital-related infection control regulations)

6. Have a history of local transmission of a local case in the past two weeks, a history of travel, a history of residence, or a history of patient contact in the area?

☐No ☐Yes (Exact date ____________________

In accordance with hospital-related infection control regulations)

7. Do you have the following physical symptoms / signs recently?

☐Fever ☐hypodynamia ☐Dry cough ☐Nasal obstruction ☐Nasal discharge

☐Diarrhea ☐Dyspnea ☐Wheezes ☐Nausea ☐Vomiting ☐None of the above

8. Before the endoscopic diagnosis and treatment, a chest CT report within the past three days should be provided on the basis of a routine examination.

If there are any related symptoms / signs mentioned above, please describe in detail (including time, inducement, severity, medication history, etc.), and further investigate

infection-related risks based on specific conditions. If necessary, we will implement related measures in accordance with hospital-related infection control regulations).

Besides, we still need to tell you that the COVID-19 has a long incubation period, strong infectivity and concealment. Although we have taken corresponding isolation and disinfection measures, you may become a highly suspected patient of the disease and need to be at home or go to the hospital for the corresponding standardized isolation observation, if any patient with confirmed or suspected 2019-nCoV infection was found in the same time as you receive endoscopic procedures.

Please fully understand the above screening contents and precautions, and promise to answer truthfully by yourself, and accept that you have the risk of potential COVID-19 infection during the endoscopic procedures:

Patient signature: Phone number:

Patient ID number: Place of residence in the past two weeks:

Screening medical staff signature: Date:
